# Exploration of High-Grade Transformation and Postoperative Radiotherapy on Prognostic Analysis for Primary Adenoid Cystic Carcinoma of the Head and Neck

**DOI:** 10.3389/fonc.2021.647172

**Published:** 2021-04-09

**Authors:** Yuelu Zhu, Xinyi Zhu, Xuemin Xue, Ye Zhang, Chunfang Hu, Wenchao Liu, Haizhen Lu

**Affiliations:** ^1^ Department of Pathology, National Cancer Center/National Clinical Research Center for Cancer/Cancer Hospital, Chinese Academy of Medical Sciences and Peking Union Medical College, Beijing, China; ^2^ Department of Radiation Oncology, National Cancer Center/National Clinical Research Center for Cancer/Cancer Hospital, Chinese Academy of Medical Sciences and Peking Union Medical College, Beijing, China

**Keywords:** adenoid cystic carcinoma, head and neck, high-grade transformation, tumor grading, postoperative radiotherapy, prognosis

## Abstract

**Background:**

Despite Adenoid cystic carcinoma (ACC) with cribriform or tubular components being recognized as a potentially indolent malignancy, ACC displaying solid or, more rarely, high-grade transformation (HGT) components is considered a more aggressive variant of the disease. As it is difficult to measure the proportion of the solid component objectively, and the role of HGT in the current grading system remains unclear, the prognostic influence of tumor grading remains controversial. In addition, postoperative radiotherapy (PORT) has been proven to be effective in local control of ACC of the head and neck (ACCHN) with a high rate of nerve invasion and close surgical margin. However it remains to be explored that whether PORT could improve the survival of patients with ACC, particularly those with HGT.

**Methods:**

A series of 73 surgically treated primary ACCHN cases were retrospectively accessed. Immunohistochemical staining was performed to observe the biphasic ductal-myoepithelial differentiation and to identify the HGT components of ACC for tumor grading. The correlation between tumor grading and clinicopathological characteristics was analyzed. Univariate and multivariate prognostic analysis were performed for progression-free survival (PFS) and overall survival (OS).

**Results:**

Of the 73 included cases, 47 were grade I-II ACC and 26 were grade III ACC. Among the grade III cases, 14 with loss of biphasic ductal-myoepithelial differentiation identified by immunostaining were classified as HGT, and could be distinguished from conventional grade III cases. These HGT cases were correlated with a high propensity of lymph node metastases and more advanced stage. Univariate analysis demonstrated that tumor grading, perineural invasion, T stage, stage groups, and PORT were predictors for PFS, whereas tumor grading, margin status, and PORT were predictors for OS. However, only tumor grading and PORT were independent predictors for PFS and OS. The patients with HGT had significantly worse prognosis than those with conventional ACC. Moreover, disease progression tended to occur more frequently in younger patients. Among the patients with HGT, those who received PORT had a longer median survival time than those who did not.

**Conclusion:**

HGT ACC identified by loss of biphasic differentiation should be considered in tumor grading. Tumor grading and PORT were independent predictors for disease progression and OS in surgically treated ACCHN patients.

## Introduction

Adenoid cystic carcinoma (ACC) is one of the most common minor salivary gland malignancies originated from the oral cavity, and is characterized by extensive invasion, frequent local recurrence, and delayed distant metastases (1). The overall 5-, 10-, and 15-year survival rates for patients with ACC of the head and neck (ACCHN) were 90.3, 79.9, and 69.2%, respectively, and radical surgery of the primary tumor has been shown to be beneficial for survival (2). Histologically, ACC is comprised of luminal ductal cells and abluminal basal/myoepithelial cells. The three major architectures of ACC are tubular, cribriform, and solid patterns. Although it is generally considered that ACC is potentially indolent, half a century ago, researchers noticed that some ACC cases presented as high grade malignancies with predominant solid morphology and rapidly worsening behavior (3–5). At present, several tumor grading systems based on histological patterns can be applied for ACC prognostic prediction (6–8). However, it is difficult to measure the proportion of solid components and to distinguish the solid and mixed components objectively. Since the concept of ACC with high-grade transformation (HGT) was formally proposed in 1999 (9), it has been debated whether HGT ACC should be regarded separately as rare cases or whether they should be integrated into the tumor grading system as a whole to evaluate their biological behavior. This study explored which method of classification would be more helpful for prognostic prediction and clinical practice. In addition, in patients with ACCHN, the surgical field involves vital organs and structures, which makes it difficult to achieve the optimal safe margin. As a result, latent residual lesions may increase the tendency of disease recurrence and decrease long-term survival. Postoperative radiotherapy (PORT) has been shown to be an effective auxiliary postoperative treatment to delay local recurrence of ACC (10). Here we further explore the effect of PORT on the survival of patients with ACC, especially those with HGT ACC.

## Materials and Methods

### Patients and Treatments

Our study comprised 73 patients with primary ACCHN who underwent surgery at National Cancer Center/National Clinical Research Center for Cancer/Cancer Hospital, Chinese Academy of Medical Sciences and Peking Union Medical College, between July 2010 and April 2018. Of the included patients, 33 were male and 40 were female. The age of the patients ranged from 21 to 76 (median, 50) years old. The ACCHN cases originated from major salivary glands (n = 17), nasal cavity and paranasal sinuses (n = 15), lip and oral cavity (n = 15), trachea (n = 9), pharynx (n = 7), external auditory canal (n = 5), larynx (n = 3), and lacrimal gland (n = 2). Twenty-nine patients received surgery alone, and 39 patients received surgery with PORT. Of these 39 patients, 28 were treated in our hospital and two patients were administrated with concurrent nimotuzumab. The most common prescription dose for tumor bed of the primary site was 60–66 Gy; the high-risk area of the clinical tumor volume was 60 Gy, and the low risk area of the clinical tumor volume was 51 Gy. The dose was increased to 70 Gy when a positive margin or gross residual disease was present. The detailed techniques and doses used for the remaining 11 patients from other hospitals were unknown. Information regarding postoperative therapy was unavailable in five patients who were lost of follow-up after surgery.

### Histopathological Reevaluation

All of the slides were reviewed by three experienced pathologists (Zhu YL, Hu CF, and Lu HZ). If there was any disagreement on diagnosis, a consensus was reached by simultaneous review using a multi-headed microscope. The tumors were categorized using the Perzin/Szanto grading system as follows (7, 11): grade I–II, with <30% solid components; and grade III, with >30% solid components. The major histopathological criteria of HGT was recommended by Seethala et al. as follows (12): 1) At least two to three times the size of grade I–II ACC nuclei; 2) fibrocellular desmoplastic stroma; 3) solid confluent nests to sheets, often filling a 40× high power field (hpf); 4) unique features, such as micropapillae or squamoid areas; 5) an incomplete abluminal cell layer and at least focally absent by immunohistochemistry; and 6) overexpression of p53. All of the tumors were staged according to AJCC 8^th^ edition (13), except the tumors of external auditory canal and trachea were staged according to the original literatures (14, 15).

### Immunohistochemical Staining

Immunohistochemical staining was performed with an immunoperoxidase technique using the automated Leica BOND-MAX machine. Positive and negative controls were included in the staining reaction, and information on the prediluted antibodies is shown in **Table 1**. The expression of biomarkers was estimated semi-quantitatively. For the biomarkers of luminal ductal cells and abluminal basal/myoepithelial cells, positive expression was defined when the proportion of positive cells was greater than 10%, and negative or focal positive expression was defined when the proportion was less than 10%. Loss of biphasic differentiation was defined in the absence of either luminal ductal cells or abluminal basal/myoepithelial cells, identified by negative or focal positive expression of corresponding biomarkers. For p53, aberrant expression was defined when the proportion of strong nuclear positive cells was greater than 60% or if the staining was completely negative. Scattered expression of p53 was defined when the intensity of staining was inconsistent (16, 17).

**Table 1:** Antibodies for immunohistochemical staining.

Antibodies	Clone	Source	Components marked
Calponin	EP63	ZhongShan-GoldenBridge, Beijing, China	Myoepithelial cells
p63	4A4	Roche Diagnostics, Shanghai, China	Myoepithelial cells
CK7	OV-TL12/30	Maxim, Fuzhou, China	Ductal cells
CK5/6	MX040	Maxim, Fuzhou, China	Ductal/Myoepithelial cells
S100	4C4.9	Maxim, Fuzhou, China	Ductal/Myoepithelial cells
p53	MX008	Maxim, Fuzhou, China	p53 protein
Ki-67	GM001	GeneTech, Shanghai, China	Proliferative antigens

### Follow-Up

The duration of progression-free survival (PFS) was measured from the day of surgery to the day of disease progression, death, or last contact (October, 2020). The duration of overall survival (OS) was measured from the day of surgery to the day of death or last contact (October, 2020). The follow-up information was collected from clinical records or *via* telephone interview. The median follow-up time was 35.25 (3–109) months and 53 (4.5–119) months for PFS and OS, respectively. Five patients were lost to follow-up after surgery.

### Statistical Analysis

All statistical analyses were performed using SPSS software (IBM SPSS Statistics, version 19). Correlations between tumor grading and clinicopathological characteristics were calculated using the χ2 test. The differences in immunophenotypes between conventional grade III cases and HGT cases were calculated by Fisher exact test. The survival curves and median survival time were generated from the Kaplan–Meier method and log rank test. Multivariate analyses were performed by forward stepwise Cox regression. P-values <0.05 were considered statistically significant.

## Result

### Clinicopathological Characteristics

Forty-seven cases were classified as grade I–II, and 26 cases were classified as grade III. PNI and close margin (<1mm) status were observed microscopically in 83.6% (n = 61) of cases and 84.9% (n = 62) of cases, respectively. Twenty patients had T1 or T2 disease, 34 patients had T3 disease, and 19 patients had T4 disease. Nine patients had lymph node metastases (LNM), and 25 patients did not. The remaining 39 patients did not receive lymph node dissection. Nineteen patients had stage I or II disease, 31 patients had stage III disease, and 23 patients had stage IV disease. The data are shown in **Table 2**.

**Table 2:** Clinicopathological characteristics.

Characteristics	n
Gender	
Male	33 (45.2)
Female	40 (54.8)
Age groups	
≤50y	37 (50.7)
>50y	36 (49.3)
Primary sites	
Major salivary	17 (23.3)
Nasal cavity and paranasal sinuses	15 (20.5)
lip and oral cavity	15 (20.5)
Trachea	9 (12.3)
Pharynx	7 (9.6)
External auditory canal	5 (6.8)
Larynx	3 (4.1)
Lacrimal gland	2 (2.7)
Tumor grading	
Grade I–II	47 (64.4)
Grade III	26 (35.6)
PNI	
Negative	12 (16.4)
Positive	61 (83.6)
Margin status	
≥1 mm	11 (15.1)
<1 mm	62 (84.9)
T stage	
1–2	20 (27.4)
3	34 (46.6)
4	19 (26)
LNM (n = 34)	
Negative	25 (73.5)
Positive	9 (26.5)
Stage groups	
I–II	19 (26)
III	31 (42.5)
IV	23 (31.5)
PORT (n = 68)	
Yes	39 (57.4)
No	29 (42.6)

PNI, perineural invasion; LNM, lymph node metastases; PORT, postoperative radiotherapy.

### Difference in Histopathological Features and Immunophenotypes Between Conventional Grade III ACC and HGT ACC

**Figure 1:**
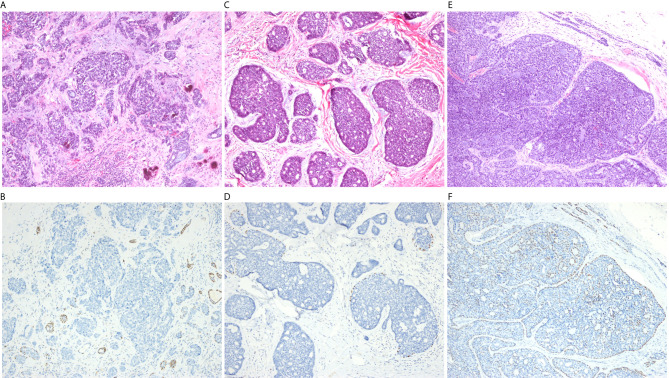
**(A)** Classic high-grade transformation (HGT) features, including severe nuclear atypia, desmoplastic stroma, and irregular solid nests (100×). **(B)** Absence of p63 staining in HGT components compared with positive staining in a few cribriform-tubular structures within the same microscope field (100×). **(C)** Non-classical HGT features include moderate nuclear atypia, myxoid/hyaline matrix, and more regular solid nests (100×). **(D)** Obvious incomplete p63 staining in a non-classic HGT case (100×). **(E)** Conventional grade III ACC usually presents as mixed cribriform and solid patterns (100×). **(F)** The p63 stained basal/myoepithelial cell layer was still within the mixed architecture of conventional grade III ACC (100×).

Immunohistochemical staining was performed in 26 grade III cases (**Table 3**). All of the 14 HGT cases were grade III, and, with the exception of one case, all lacked basal/myoepithelial cells, as defined by negative or focal expression of p63. As the exception, one case had weak staining of p63 in the squamoid area of the HGT components. Seven of the HGT cases exhibited classic HGT features, including severe nuclear atypia, desmoplastic stroma, expanded solid nests, and loss of p63 staining for abluminal basal/myoepithelial cells (**Figures 1A, B**). The remaining seven HGT cases exhibited moderate nuclear atypia, myxoid/hyaline matrix, small solid nests, and loss of p63 staining for abluminal basal/myoepithelial cells (**Figures 1C, D**). Aberrant expression of p53 existed in four of the 14 HGT case. Ki-67 index was greater than 20% in 9 (64.3%) of the total HGT cases. The remaining 12 conventional grade III cases still showed obvious cribriform components mixed with solid areas, basaloid cells lacking cytoplasm, and the presence of a p63 stained basal/myoepithelial cell layer (**Figures 1E, F**). All of the conventional grade III cases had scattered expression of p53. The Ki-67 index was greater than 20% in only 1 (8.3%) of these cases. Positive CK7 expression was detected in all of the 26 cases, and there was no significant difference in the expression of CK5/6 and S100 between conventional grade III cases and HGT cases. The summarized histopathological features and immunophenotypes are shown in **Supplementary Material 1**.

**Table 3:** Differences in immunophenotypes between conventional grade III and HGT ACC.

Antibodies	n	Conventional grade III	HGT	P-value
Calponin				0.008
Neg/f Pos	12 (46.2)	2 (16.7)	10 (71.4)	
Pos	14 (53.8)	10 (83.3)	4 (28.6)	
p63				<0.001
Neg/f Pos	13 (50)	0 (0)	13 (92.9)	
Pos	13 (50)	12 (100)	1 (7.1)	
CK7				–
Neg/f Pos	0 (0)	0 (0)	0 (0)	
Pos	26 (100)	12 (100)	14 (100)	
CK5/6				0.483
Neg/f Pos	2 (7.7)	0 (0)	2 (14.3)	
Pos	24 (92.3)	12 (100)	12 (85.7)	
S100				0.683
Neg/f Pos	9 (34.6)	5 (41.7)	4 (28.6)	
Pos	17 (65.4)	7 (58.3)	10 (71.4)	
p53				0.100
Scattered	22 (84.6)	12 (100)	10 (71.4)	
Aberrant	4 (5.4)	0 (0)	4 (28.6)	
Ki-67				0.005
≤20%	16 (61.5)	11 (91.7)	5 (35.7)	
>20%	10 (38.5)	1 (8.3)	9 (64.3)	

HGT, high-grade transformation; ACC, adenoid cystic carcinoma; Neg, negative; f, focal; Pos, positive.

### Correlation Between Tumor Grading and Clinicopathological Characteristics

Tumor grading was correlated with LNM (P = 0.009) and stage groups (P = 0.039), but was not correlated with sex, age groups, primary sites, PNI, margin status, T stage, or PORT (**Table 4**). Among the 34 patients with lymph node dissection, 13% (3/23) of patients with grade I–II had LNM, 25% (1/4) of patients with conventional grade III had LNM, and 71.4% (5/7) of patients with HGT had LNM. Among all cases, 70.2% (33/47) of patients with grade I–II, 66.6% (8/12) of patients with conventional grade III, and 92.9% (13/14) of patients with HGT had stage III–IV disease.

**Table 4:** Correlation between tumor grading and clinicopathological characteristics.

Characteristics	n	Grade I–II	Conventional grade III	HGT	P-value
Gender					0.15
Male	33 (45.2)	21 (44.7)	8 (66.7)	4 (28.6)	
Female	40 (54.8)	26 (55.3)	4 (33.3)	10 (71.4)	
Age groups					0.566
≤50y	37 (50.7)	26 (55.3)	5 (41.7)	6 (42.9)	
>50y	36 (49.3)	21 (44.7)	7 (58.3)	8 (57.1)	
Primary sites					0.641
Major salivary	17 (23.3)	7 (14.9)	4 (33.3)	6 (42.9)	
Nasal cavity and paranasal sinuses	15 (20.5)	10 (21.3)	2 (16.7)	3 (21.4)	
lip and oral cavity	15 (20.5)	10 (21.3)	2 (16.7)	3 (21.4)	
Trachea	9 (12.3)	7 (14.9)	2 (16.7)	0 (0)	
Pharynx	7 (9.6)	6 (12.8)	1 (8.3)	0 (0)	
External auditory canal	5 (6.8)	3 (6.4)	1 (8.3)	1 (7.1)	
Larynx	3 (4.1)	3 (6.4)	0 (0)	0 (0)	
Lacrimal gland	2 (2.7)	1 (2.1)	0 (0)	1 (7.1)	
PNI					0.169
Negative	12 (16.4)	10 (21.3)	2 (16.7)	0 (0)	
Positive	61 (83.6)	37 (78.7)	10 (83.3)	14 (100)	
Margin status					0.422
≥1 mm	11 (15.1)	9 (19.1)	1 (8.3)	1 (7.1)	
<1 mm	62 (84.9)	38 (80.9)	11 (91.7)	13 (92.9)	
T stage					0.127
1–2	20 (27.4)	14 (29.8)	4 (33.3)	2 (14.3)	
3	34 (46.6)	25 (53.2)	4 (33.3)	5 (35.7)	
4	19 (26)	8 (17)	4 (33.3)	7 (50)	
LNM (n = 34)					0.009
Negative	25 (73.5)	20 (87)	3 (75)	2 (28.6)	
Positive	9 (26.5)	3 (13)	1 (25)	5 (71.4)	
Stage groups					0.039
I–II	19 (26)	14 (29.8)	4 (33.3)	1 (7.1)	
III	31 (42.5)	23 (48.9)	4 (33.3)	4 (28.6)	
IV	23 (31.5)	10 (21.3)	4 (33.3)	9 (64.3)	
PORT (n = 68)					0.664
Yes	39 (57.4)	27 (61.4)	6 (50)	6 (50)	
No	29 (42.6)	17 (38.6)	6 (50)	6 (50)	

HGT, high-grade transformation; PNI, perineural invasion; LNM, lymph node metastasis; PORT, postoperative radiotherapy.

### Survival Analysis

**Figure 2:**
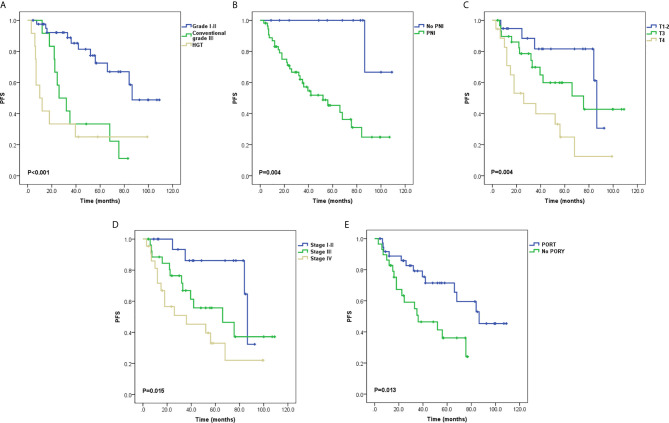
Survival curves for progression-free survival (PFS) **(A)** Tumor grading, **(B)** Perineural invasion (PNI), **(C)** T stage, **(D)** Stage groups, and **(E)** Postoperative radiotherapy (PORT).

**Figure 3:**
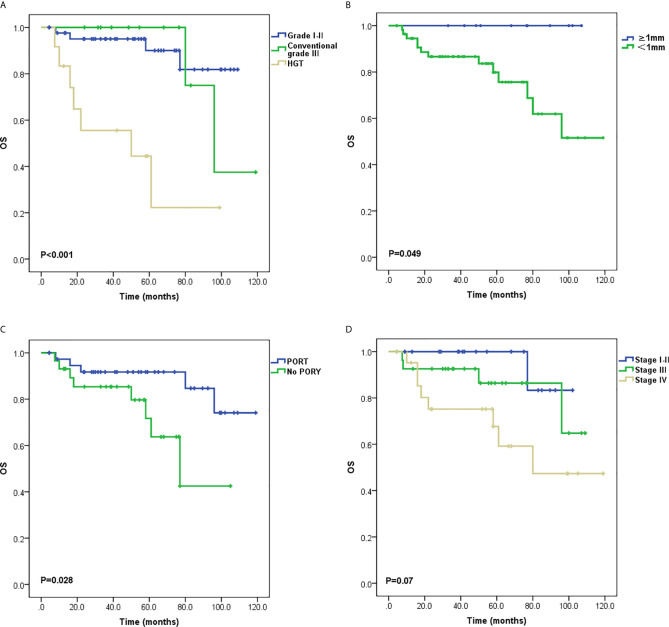
Survival curves for overall survival (OS) **(A)** Tumor grading, **(B)** Margin status, **(C)** Postoperative radiotherapy (PORT) and **(D)** Stage groups.

Local recurrence occurred in 11 patients, two of whom also had sternum metastasis and LNM, and three also had lung metastases. Distant metastasis was the only progressive event in 19 patients. The most common metastatic site was the lung, and other sites included the parietal bone, dura meter, liver, chest wall, and cervical subcutaneous tissue. Univariate analysis of PFS demonstrated that tumor grading (P < 0.001; **Figure 2A**), PNI (P = 0.004; **Figure 2B**), T stage (P = 0.004; **Figure 2C**), stage groups (P = 0.015; **Figure 2D**), and PORT (P = 0.013; **Figure 2E**) were significant factors. Death occurred in 13 patients. Univariate analysis of OS demonstrated that tumor grading (P < 0.001; **Figure 3A**), margin status (P = 0.049; **Figure 3B**), and PORT (P = 0.028; **Figure 3C**) were significant factors. Patients with more advanced stage had worse outcomes, although this result was not significant (P = 0.07; **Figure 3D**). The comparison of the median survival time among different variables for PFS and OS is shown in **Table 5**. All of the 11 patients with a distance greater than 1 mm from the surgical margin were alive at the end of follow-up, whereas 13 of the 53 patients with distance less than 1 mm from the surgical margin were dead at the end of follow-up. As a result, the median OS time of the margin status could not be calculated.

**Table 5:** Comparison of the median survival time among prognostic predictors on univariate analyses.

Characteristics	PFS		OS	
	Median for survival time	P-value	Median for survival time	P-value
Tumor grading		<0.001		<0.001
Grade I–II	86.5		Not reached	
Conventional grade III	26		96	
HGT	10		50	
PNI		0.004		–
Negative	Not reached		–	
Positive	52		–	
Margin status		–		0.049
≥1 mm	–		cannot be calculated*	
<1 mm	–		cannot be calculated*	
T stage		0.004		–
1–2	86.5		–	
3	75.5		–	
4	26		–	
Stage groups		0.015		0.07
I–II	86.5		Not reached	
III	66		Not reached	
IV	36		80	
PORT		0.013		0.028
Yes	86.5		Not reached	
No	36		77	

PFS, progression-free survival; OS, overall survival; HGT, high-grade transformation; PNI, perineural invasion; PORT, postoperative radiotherapy. *All of the patients with a surgical margin ≥1 mm survived, and all of the deaths occurred in patients with a surgical margin <1 mm; thus, the median overall survival time cannot be calculated.

Multivariate analysis of PFS demonstrated that tumor grading, age groups, and PORT were independent factors. With regard to grade I–II cases, the hazard ratio (HR) of conventional grade III cases was 5.035 (95% confidence interval [CI] = 1.979–12.814, P = 0.001), while that of HGT cases was 9.616 (95% CI = 3.222–28.697, P < 0.001). With regard to patients aged ≤50 years, the HR of patients >50 years old was 0.321 (95% CI = 0.138–0.747, P = 0.008). With regard to patients who received PORT, the HR of patients who did not was 3.895 (95% CI = 1.636–9.273, P = 0.002). Multivariate analysis of OS demonstrated that only tumor grading and PORT were independent factors. With regard to grade I–II cases, the HR of conventional grade III cases was 1.77 (95% CI = 0.318–9.849, P = 0.514), while that of HGT cases was 10.728 (95% CI = 2.998–38.393, P < 0.001). With regard to patients who received PORT, the HR of patients who did not was 4.336 (95% CI = 1.214–15.489, P = 0.024). The data of multivariate analysis is shown in **Table 6**.

**Table 6:** Multivariate Cox regression analysis.

Characteristics	PFS			OS		
	HR	95% CI	P-value	HR	95% CI	P-value
Age groups			0.008			–
≤50y (reference)	1			–	–	
>50y	0.321	0.138–0.747		–	–	
PNI			0.066			–
Negative (reference)	1			–	–	
Positive	6.698	0.88–51.002		–	–	
Tumor grading			<0.001			0.001
Grade I–II (reference)	1			1		
Conventional Grade III	5.035	1.979–12.814	0.001	1.77	0.318–9.849	0.514
HGT	9.616	3.222–28.697	<0.001	10.728	2.998–38.393	<0.001
PORT			0.002			0.024
Yes (reference)	1			1		
No	3.895	1.636–9.273		4.336	1.214–15.489	

PFS, progression-free survival; OS, overall survival; HR, hazard ratio; CI, confidence interval; HGT, high-grade transformation; PORT, postoperative radiotherapy.

In addition, for the six HGT patients who received PORT, the median PFS was 39.5 months, whereas the median OS was not reached. For the six HGT patients who did not receive PORT, the median PFS was 7 months, whereas the median OS was 18 months (**Table 7**).

**Table 7:** Comparison of the median survival time between patients with HGT and PORT and patients with HGT and no PORT.

Characteristics	PFS			OS	
	Median for survival time	P-value		Median for survival time	P-value
PORT		0.037			0.094
Yes	39.5			Not reached	
No	7			18	

HGT, high-grade transformation; PORT, postoperative radiotherapy; PFS, progression-free survival; OS, overall survival.

## Discussion

ACC is a biphasic ductal-myoepithelial differentiated malignant tumor which usually appears in a mixed form. The major histological structures include tubular, cribriform, and solid patterns. Spiro et al. (6) graded ACC histologically according to whether the solid area of the tumor was greater than 50%. Their subsequent study revealed that staging had a greater impact on prognosis than histological grading (18). Another more widely used grading system was based on whether the solid components of ACC were greater than 30% (7, 11). A later study demonstrated the prognostic significance of tumor grading was independent of staging (19). However, some scholars believe that the grading method based on the proportion of solid components is arbitrary (20). Moreover, in clinical practice, the histological features of ACC usually present as complex architectures with more than one pattern. In this situation, whether solid components that coexist with tubular or cribriform patterns indicate a worse prognosis need to be further explored (21). Weert et al. (8) found that the existence of any solid component in ACC was an adverse prognostic factor. Later studies also found that compared with solid components greater than 30%, the presence of solid components was an independent prognostic factor of recurrence-free-survival and OS (22, 23). In our opinion, neither the presence nor the proportion of solid components reflected the lethality of the most aggressive components of the tumor on patient survival. Although most ACC cases are indolent and have delayed mortality, if patients with highly aggressive components could be distinguished from those with indolent tumor at an early stage, more attention and appropriate treatment strategies could be given. This may have significant implications in the prognosis of these patients.

The most aggressive components of ACC are generally considered to be the HGT components, as a synonym of anaplasia or dedifferentiation, which was first formally described in ACC by Cheuk et al. (9). Later, Seethala et al. (12) summarized the histopathological characteristics from 11 HGT cases in detail. However, in view of the complexity of diagnostic criteria emphasized by the subjective descriptive features, very few cases are likely to satisfy the diagnosis of HGT. Indeed, most previous studies have been case reports or small series (24). In a large series with 135 cases of salivary gland ACC, there were only 16 cases with HGT (22). Even in this study, no statistical difference in LNM and prognosis of HGT ACC could be obtained due to the small number of cases. Since ACC is generally considered to originate from intercalated duct, the histological origin determines biphasic ductal-myoepithelial differentiated characteristic of ACC. We believe that the diagnostic essentials of HGT should be focused on the loss of biphasic differentiation of ACC. Both Cheuk et al. (9) and Seethala et al. (12) emphasized the absence of a basal/myoepithelial cell layer in HGT diagnosis. This may be attributed to the belief that overgrowth of ductal components rather than myoepithelial components leads to lethal events. In this series, there were 26 cases of grade III, 14 of which were recognized by loss of biphasic differentiation, represented by the absence of basal/myoepithelial cell immunostaining. Although one of the cases had weak p63 staining in the squamoid area of the tumor, this expression of p63 was not abluminal-staining pattern. Besides squamoid area is regarded as one kind of unique features of HGT components according to the HGT criteria. Therefore the expression of p63 in this case did not affect the diagnosis of basal/myoepithelial cell absence.

Seethala et al. (12) recommended that HGT be diagnosed as the presence of at least three of the major criteria. Of the 14 cases, seven met the rigorous criteria, whereas the remaining seven met less than three of the major criteria. Although these seven cases do not completely fulfill the major criteria for HGT, we still insist that all the 14 cases should be classified as HGT ACC. First of all, previous studies have outlined that transformed components exist in HGT ACC cases (9, 12, 25). This morphological transition may be evidence that gradual loss of basal/myoepithelial cell differentiation occurs during the HGT process. This histological transition could also be observed in our cases (**Figure 1**). Furthermore, the LNM rate of the HGT group was significantly higher than that of the other groups (P = 0.009). Besides, the Ki-67 index of the HGT cases was higher than that of the conventional grade III cases (P = 0.005), indicating that the HGT cases had higher proliferative activity. More importantly, although there was no significant prognostic difference between HGT cases and conventional grade III cases with regard to PFS, there was in terms of OS. Indeed, only two of the 12 conventional grade III patients died during the 80–99 months after surgery, whereas seven of the 14 HGT patients died during the 7.5–61 months after surgery (**Supplementary Material 2**). For patients with conventional grade III and HGT, the median OS was 99 months and 50 months, respectively. The OS curve showed a significant difference between the HGT group and the conventional grade III group. The data from Cox stratification analysis of OS also showed that there was no significant difference between patients with conventional grade III and those with grade I–II, whereas the risk of death in patients with HGT was much higher than in those with grade I–II (HR = 10.728, P < 0.001). These results support our point of view that loss of basal/myoepithelial cell differentiation is the most important criterion of HGT. The absence of the myoepithelial cell layer in ACC probably indicates ductal cell overgrowth of the tumor accompanied by more aggressive behavior. As a result, patients with HGT tumors in our series account for 19% of the primary ACCHN cases. Interestingly, a previous study from Fordice et al. (26) considered solid features as fulfilling two criteria: >10% of solid components, and the presence of an anaplastic area within the solid architecture. Their study confirmed the adverse impact of solid features in univariate prognostic analysis. Indeed, the anaplasia of ACC is equivalent to the HGT components. In view of the finding from a previous meta-analysis that a higher LNM rate correlates with solid or higher grade ACC (27), it may be that the unidentified HGT ACC tumors also play a crucial role. Our study used basal/myoepithelial cell biomarkers to identify whether the solid area possess biphasic differentiation or not, and further confirmed the predictive role of tumor grading independent of staging under the premise of HGT ACC identification.

In our study, although age groups were not statistically significant in univariate analysis, younger patients had worse prognosis in terms of PFS in multivariate analysis. Among the 14 patients with HGT ACC, six were ≤50 years old and eight were >50 years old. Given that the age groups were not correlated with tumor grading in our study, the finding that HGT components are more likely to occur in the elderly as described in previous literatures (9, 12) may not hold true for all cases. In our study, T4 or stage IV disease was slightly more common in younger patients than older patients (**Supplementary Material 2**). Both T stage and stage groups were significant on PFS univariate analysis. This more advanced stage might lead to the worse prognosis of the younger patients, despite no independent prognostic significance on the staging factors themselves. Besides, our study revealed no correlation between primary sites and tumor grading. We found that HGT ACC could originate from superficial sites such as the submandibular gland, and was associated with LNM and poor outcome. Although the surgical margin was significant on OS univariate analysis, it was not an independent factor. In our study, the rates of close margin status and PNI were 84.9 and 83.6%, respectively. We agree with the previously held opinion that a sufficient safe margin of ACCHN is difficult to achieve due to the infiltrative and perineural characteristics of this disease (10, 22).

Radical surgery and PORT are the standard treatments for patients with high-risk factors such as advanced stage, nerve invasion, or residual tumor (28). Safina et al. indicated that the 10-year local recurrence survival rate of patients with PORT and no PORT was 90.1 and 41.6%, respectively (29). A multi-center retrospective study in Japan confirmed that sufficient radiation therapy (≥60 Gy) was beneficial for OS and was effective for local control (30). Moreover, Stefano et al. (31) revealed that the prognosis of patients with salivary ACC with lung metastases was better than that of patients with metastases to the liver, bone, and other sites. Although PORT could not prevent distant metastases, for patients with subclinical distant metastases, Chen et al. believed that better local control will be important to delay disease progression and maintain quality of life (32). Given that the ACCHN patients in this retrospective study had higher rates of PNI and close margin status, it was expected that PORT should be employed in more patients. However, only 57.4% patients received PORT, which might be due to the low acceptance of PORT from some patients in this series. Our study confirmed that patients who received PORT obtained significant benefits, both in terms of disease progression and OS, compared with patients who did not. However, there remains further work to be done to improve patients’ acceptance of PORT. Recent studies revealed that carbon ion radiotherapy assisted by treatment planning software such as a raster-scanning system could decrease the complication rate and elevate treatment efficiency (28, 33), which shows promise for future treatment strategies. Although patients with HGT had worse prognosis in our study, those who received PORT had a longer median survival time than those who did not (**Table 7**). Moreover, among the six patients with HGT who did not receive PORT, one suffered from tumor recurrence 3 months after surgery. This patient received PORT after removal of the recurrent lesion and survived a total of 61 months after the initial operation. Another patient with HGT received chemotherapy at the local hospital as a result of sternal metastasis that occurred 7 months after surgery; and survived a total of 50 months after surgery. Deaths occurred within 2 years of surgery in the remaining four patients with HGT who did not receive any adjuvant therapy. Several HGT ACC case reports also showed that patients who received PORT or combined chemo-radiotherapy had no evidence of disease during a follow-up ranging from 5 to 36 months (34–37). Thus, it seems that active postoperative therapy may bring hope to patients who are believed to have poor outcomes. It is though that the loss of basal/myoepithelial differentiation might bring the biological characteristics of ACC closer to those of some high grade carcinomas such as salivary ductal carcinoma, which may be more sensitive to adjuvant therapy. Therefore, research on precise therapy should be explored in patients with ACC with different histopathological grades for long-term survival benefits.

## Conclusion

Loss of biphasic differentiation as identified by the absence of basal/myoepithelial cells is the most important diagnostic criterion of HGT ACC. PORT, and tumor grading system including HGT had significant implications on prognosis of surgically treated patients with primary ACCHN. As this is a retrospective study from a single center, further studies should be performed on appropriate therapeutic strategies for patients with different tumor grades.

## Data Availability Statement

The original contributions presented in the study are included in the article/**Supplementary Material**. Further inquiries can be directed to the corresponding author.

## Ethics Statement

The studies involving human participants were reviewed and approved by National GCP Center for Anticancer Drugs, The Independent Ethics Committee. Written informed consent for participation was not required for this study in accordance with the national legislation and the institutional requirements.

## Author Contributions

HL and YLZ designed this study. XZ, YLZ, and CH enrolled the patients and collected the clinical data. YLZ, CH, and HL reviewed the pathological sections. YLZ, XX, and WL conducted the statistical analyses. The manuscript was drafted by YLZ and XZ. All authors participated in interpretation of the results. All authors contributed to the article and approved the submitted version.

## Funding

This study was supported by Beijing Hope Run Special Fund of Cancer Foundation of China, No. LC2018A19.

## Conflict of Interest

The authors declare that the research was conducted in the absence of any commercial or financial relationships that could be construed as a potential conflict of interest.

## References

[1] Jaso J Malhotra R. Adenoid cystic carcinoma. Arch Pathol Lab Med (2011) 135(4):511–5. 10.1043/2009-0527-RS.1 21466371

[2] Ellington CL Goodman M Kono SA Grist W Wadsworth T Chen AY. Adenoid cystic carcinoma of the head and neck: Incidence and survival trends based on 1973-2007 Surveillance, Epidemiology, and End Results data. Cancer (2012) 118(18):4444–51. 10.1002/cncr.27408 22294420

[3] Patey DH Thackray AC. The treatment of parotid tumours in the light of a pathological study of parotidectomy material. Br J Surg (1958) 45(193):477–87. 10.1002/bjs.18004519314 13536351

[4] Stewart J. Carcinoma of salivary glands showing the cylindroma pattern. Br J Surg (1961) 49:241–5. 10.1002/bjs.18004921502 13917137

[5] Eby LS Johnson DS Baker HW. Adenoid cystic carcinoma of the head and neck. Cancer (1972) 29(5):1160–8. 10.1002/1097-0142(197205)29:5<1160::aid-cncr2820290506>3.0.co;2-1 4336629

[6] Spiro RH Huvos AG Strong EW. Adenoid cystic carcinoma of salivary origin. A clinicopathologic study of 242 cases. Am J Surg (1974) 128(4):512–20. 10.1016/0002-9610(74)90265-7 4371368

[7] Szanto PA Luna MA Tortoledo ME White RA. Histologic grading of adenoid cystic carcinoma of the salivary glands. Cancer (1984) 54(6):1062–9. 10.1002/1097-0142(19840915)54:6<1062::aid-cncr2820540622>3.0.co;2-e 6088017

[8] Van Weert S Van Der Waal I Witte BI Leemans CR Bloemena E. Histopathological grading of adenoid cystic carcinoma of the head and neck: analysis of currently used grading systems and proposal for a simplified grading scheme. Oral Oncol (2015) 51(1):71–6. 10.1016/j.oraloncology.2014.10.007 25456010

[9] Cheuk W Chan JK Ngan RK. Dedifferentiation in adenoid cystic carcinoma of salivary gland: an uncommon complication associated with an accelerated clinical course. Am J Surg Pathol (1999) 23(4):465–72. 10.1097/00000478-199904000-00012 10199477

[10] Coca-Pelaz A Rodrigo JP Bradley PJ Vander Poorten V Triantafyllou A Hunt JL. Adenoid cystic carcinoma of the head and neck–An update. Oral Oncol (2015) 51(7):652–61. 10.1016/j.oraloncology.2015.04.005 25943783

[11] Perzin KH Gullane P Clairmont AC. Adenoid cystic carcinomas arising in salivary glands: a correlation of histologic features and clinical course. Cancer (1978) 42(1):265–82. 10.1002/1097-0142(197807)42:1<265::aid-cncr2820420141>3.0.co;2-z 208752

[12] Seethala RR Hunt JL Baloch ZW Livolsi VA Leon Barnes E. Adenoid cystic carcinoma with high-grade transformation: a report of 11 cases and a review of the literature. Am J Surg Pathol (2007) 31(11):1683–94. 10.1097/PAS.0b013e3180dc928c 18059225

[13] Ei-Naggar AK Chan JKC Grandis JR Takata T Slootweg PJ. WHO Classification of Head and Neck Tumors. Lyon: IARC (2017).

[14] Morita S Mizumachi T Nakamaru Y Sakashita T Kano S Hoshino K. Comparison of the University of Pittsburgh staging system and the eighth edition of the American Joint Committee on Cancer TNM classification for the prognostic evaluation of external auditory canal cancer. Int J Clin Oncol (2018) 23(6):1029–37. 10.1007/s10147-018-1314-3 29974295

[15] Hogerle BA Lasitschka F Muley T Bougatf N Herfarth K Adeberg S. Primary adenoid cystic carcinoma of the trachea: clinical outcome of 38 patients after interdisciplinary treatment in a single institution. Radiat Oncol (2019) 14(1):117. 10.1186/s13014-019-1323-z 31272473 PMC6610895

[16] Yemelyanova A Vang R Kshirsagar M Lu D Marks MA Shih Ie M. Immunohistochemical staining patterns of p53 can serve as a surrogate marker for TP53 mutations in ovarian carcinoma: an immunohistochemical and nucleotide sequencing analysis. Mod Pathol (2011) 24(9):1248–53. 10.1038/modpathol.2011.85 21552211

[17] Ando K Oki E Saeki H Yan Z Tsuda Y Hidaka G. Discrimination of p53 immunohistochemistry-positive tumors by its staining pattern in gastric cancer. Cancer Med (2015) 4(1):75–83. 10.1002/cam4.346 25354498 PMC4312120

[18] Spiro RH Huvos AG. Stage means more than grade in adenoid cystic carcinoma. Am J Surg (1992) 164(6):623–8. 10.1016/s0002-9610(05)80721-4 1334380

[19] Zhang CY Xia RH Han J Wang BS Tian WD Zhong LP. Adenoid cystic carcinoma of the head and neck: clinicopathologic analysis of 218 cases in a Chinese population. Oral Surg Oral Med Oral Pathol Oral Radiol (2013) 115(3):368–75. 10.1016/j.oooo.2012.11.018 23453028

[20] Seethala RR. Histologic grading and prognostic biomarkers in salivary gland carcinomas. Adv Anat Pathol (2011) 18(1):29–45. 10.1097/PAP.0b013e318202645a 21169736

[21] Batsakis JG Luna MA El-Naggar A. Histopathologic grading of salivary gland neoplasms: III. Adenoid cystic carcinomas. Ann Otol Rhinol Laryngol (1990) 99(12):1007–9. 10.1177/000348949009901215 2173892

[22] Xu B Drill E Ho A Ho A Dunn L Prieto-Granada CN. Predictors of Outcome in Adenoid Cystic Carcinoma of Salivary Glands: A Clinicopathologic Study With Correlation Between MYB Fusion and Protein Expression. Am J Surg Pathol (2017) 41(10):1422–32. 10.1097/PAS.0000000000000918 PMC5597477 28719465

[23] Mays AC Hanna EY Ferrarotto R Phan J Bell D Silver N. Prognostic factors and survival in adenoid cystic carcinoma of the sinonasal cavity. Head Neck (2018) 40(12):2596–605. 10.1002/hed.25335 30447126

[24] Dutta A Arun P Arun I. Adenoid Cystic Carcinoma with Transformation to High Grade Carcinomatous and Sarcomatoid Components: A Rare Case Report with Review of Literature. Head Neck Pathol (2020) 14(4):1094–104. 10.1007/s12105-019-01120-3 PMC7669942 31898057

[25] Nagao T Gaffey TA Serizawa H Sugano I Ishida Y Yamazaki K. Dedifferentiated adenoid cystic carcinoma: a clinicopathologic study of 6 cases. Mod Pathol (2003) 16(12):1265–72. 10.1097/01.MP.0000097366.88165.08 14681328

[26] Fordice J Kershaw C El-Naggar A Goepfert H. Adenoid cystic carcinoma of the head and neck: predictors of morbidity and mortality. Arch Otolaryngol Head Neck Surg (1999) 125(2):149–52. 10.1001/archotol.125.2.149 10037280

[27] Martins-Andrade B Dos Santos Costa SF Sant’ana MSP Altemani A Vargas PA Fregnani ER. Prognostic importance of the lymphovascular invasion in head and neck adenoid cystic carcinoma: A systematic review and meta-analysis. Oral Oncol (2019) 93:52–8. 10.1016/j.oraloncology.2019.04.014 31109696

[28] Jensen AD Poulakis M Nikoghosyan AV Welzel T Uhl M Federspil PA. High-LET radiotherapy for adenoid cystic carcinoma of the head and neck: 15 years’ experience with raster-scanned carbon ion therapy. Radiother Oncol (2016) 118(2):272–80. 10.1016/j.radonc.2015.05.010 26164774

[29] Ali S Palmer FL Katabi N Lee N Shah JP Patel SG. Long-term local control rates of patients with adenoid cystic carcinoma of the head and neck managed by surgery and postoperative radiation. Laryngoscope (2017) 127(10):2265–9. 10.1002/lary.26565 PMC5661868 28322454

[30] Takebayashi S Shinohara S Tamaki H Tateya I Kitamura M Mizuta M. Adenoid cystic carcinoma of the head and neck: a retrospective multicenter study. Acta Otolaryngol (2018) 138(1):73–9. 10.1080/00016489.2017.1371329 28899226

[31] Cavalieri S Mariani L Vander Poorten V Van Breda L Cau MC Lo Vullo S. Prognostic nomogram in patients with metastatic adenoid cystic carcinoma of the salivary glands. Eur J Cancer (2020) 136:35–42. 10.1016/j.ejca.2020.05.013 32629365

[32] Chen AM Bucci MK Weinberg V Garcia J Quivey JM Schechter NR. Adenoid cystic carcinoma of the head and neck treated by surgery with or without postoperative radiation therapy: prognostic features of recurrence. Int J Radiat Oncol Biol Phys (2006) 66(1):152–9. 10.1016/j.ijrobp.2006.04.014 16904520

[33] Sulaiman NS Demizu Y Koto M Saitoh JI Suefuji H Tsuji H. Multicenter Study of Carbon-Ion Radiation Therapy for Adenoid Cystic Carcinoma of the Head and Neck: Subanalysis of the Japan Carbon-Ion Radiation Oncology Study Group (J-CROS) Study (1402 HN). Int J Radiat Oncol Biol Phys (2018) 100(3):639–46. 10.1016/j.ijrobp.2017.11.010 29413278

[34] Malhotra KP Agrawal V Pandey R. High grade transformation in adenoid cystic carcinoma of the parotid: report of a case with cytologic, histologic and immunohistochemical study. Head Neck Pathol (2009) 3(4):310–4. 10.1007/s12105-009-0122-5 PMC2791488 20016788

[35] Ly CK Cheng HM Vermeulen T. High grade transformation in a case of adenoid cystic carcinoma associated with Epstein-Barr virus expression. Pathology (2013) 45(7):693–5. 10.1097/PAT.0000000000000012 24247629

[36] Sayar H Sarioglu S Bakaris S Yildirim I Oztarakci H. High-grade transformation of adenoid cystic carcinoma delineated with a fibrous rim: a case report. Balkan Med J (2013) 30(3):333–6. 10.5152/balkanmedj.2013.7220 PMC4115909 25207133

[37] Boon IS Warfield AT Ahmed SK Boon CS Hartley A. Dedifferentiated adenoid cystic carcinoma of the nasopharynx: a rare entity of head and neck cancer. BMJ Case Rep (2016) 2016. 10.1136/bcr-2016-215889 PMC4956971 27402588

